# Stakeholder input on the CAHPS ambulatory surveys

**DOI:** 10.1186/s41687-025-00983-1

**Published:** 2025-12-22

**Authors:** Ron D. Hays, Julie A. Brown, Emma Bianculli, Marc Elliott

**Affiliations:** 1 RAND Corporation, Santa Monica, CA USA; 2 University of California, Los Angeles, CA USA; 3 UCLA Department of Medicine, 1100 Glendon Avenue, Los Angeles, CA 90024 USA

**Keywords:** CAHPS^®^, ExpertLens™, Stakeholder input, Patient experience, Ambulatory care

## Abstract

**Background:**

The Consumer Assessment of Healthcare Providers and Systems (CAHPS^®^) surveys are widely used to evaluate patients’ experiences with healthcare. Although the surveys have been extensively assessed and periodically updated, concerns persist regarding their content, length, and score distributions. This study aimed to gather systematic stakeholder feedback to inform future revisions of CAHPS ambulatory surveys.

**Methodology:**

A modified Delphi method was employed using the ExpertLens™ online platform. A panel of 20 members representing a broad stakeholder community, including survey sponsors, survey experts, patient experience advocates, and federal representatives, participated in three phases. The first phase was an initial rating of the essentialness (required, optional, not essential) of 46 existing item topics using a 1 (Not Essential) to 9 (Very Essential) scale with scores of 1–3 used for a topic that should not be included, 4–6 used for a topic that should be optional, and 7–9 for a topic that should be required in a CAHPS survey of health plans, clinicians, or group practices. The second phase was an asynchronous online discussion of the initial ratings, and the third phase was a final rating of the 46 existing item topics. The reliability of ratings was assessed using a mixed-effects analysis of variance model. Means and standard deviations of essentialness ratings were also analyzed. Verbatim comments from the experts were summarized to provide additional insights.

**Results:**

Reliability of expert 1–9 essentialness ratings improved from the initial round (reliability = 0.63, intraclass correlation = 0.08) to the final round (reliability = 0.70, intraclass correlation = 0.10). While most existing items were deemed essential by most stakeholders, there were noteworthy (0.08 or larger) increases from the initial to final rating phases in essentialness for items related to digital access, medication reconciliation, provider communication, and appeals processes, and notable decreases for specialist care ratings, access to medical questions during off-hours, and provider knowledge of chronic conditions. Stakeholders emphasized the importance of access to care, communication and coordination, respectful interactions with staff and providers, and clear cost information. Several potential topics missing from current surveys were identified, including unfair treatment, mental health integration, maternity care, language concordance, trust, self-management, patient safety, continuity of care, care coordination, and claims processing.

**Conclusions:**

This study provides valuable insights into stakeholder perspectives on the relevance and potential improvements to CAHPS ambulatory survey content. The findings support revisions to existing items to enhance their clarity and actionability, as well as the inclusion of new topics that reflect evolving healthcare priorities and patient needs. The identified areas for expansion offer opportunities to create more comprehensive and impactful assessments of patient experiences in ambulatory care settings.

**Supplementary Information:**

The online version contains supplementary material available at 10.1186/s41687-025-00983-1.

## Background

The Consumer Assessment of Healthcare Providers and Systems (CAHPS^®^) Health Plan and Clinician and Group surveys are developed using a rigorous multi-step process that includes qualitative and quantitative methods [1–11]. The development included a public call for measures, an extensive environmental scan, focus groups with English and Spanish-language patients, cognitive testing with English and Spanish-language patients, informal input from stakeholders, and multiple field tests in both English and Spanish.

The CAHPS surveys are periodically revised to incorporate changes in healthcare delivery and insights gained from previous administrations. Key updates include the releases of the Health Plan Survey 5.0 (2012) and 5.1 (2020), and the Clinician and Group Survey 3.0 (2015) and 3.1 (2020), with the latter versions of both surveys incorporating telehealth and in-person visits.

The National Advisory Council of the Agency for Healthcare Research and Quality (AHRQ) and some researchers (e.g., Bland et al. [12]) have raised concerns such as survey content, length, and score distributions. This paper summarizes the stakeholder feedback and proposes recommendations for future CAHPS ambulatory survey content. In response to the calls for updating the CAHPS ambulatory surveys, a modified Delphi method [13] was used to collect systematic feedback from stakeholders regarding potential revisions to the surveys. Our objectives were to gather input from a diverse group of stakeholders on two key areas: (1) revisions or removal of existing survey items and (2) suggestions for new item content.

## Methods

### Sample

Thirty potential Technical Expert Panel (TEP) members were identified to represent key stakeholder groups: CAHPS survey sponsors, patient experience advocates, survey experts, and federal stakeholders. CAHPS survey sponsors include employers, health plans, quality improvement organizations, and non-profit organizations. Patient experience advocates are people with extensive knowledge about patient experience with care. Survey experts are those who specialize in survey methodology. Federal stakeholders include people in leadership positions at federal agencies.

Initial outreach was conducted via email, and then a phone follow-up. Appendix A displays the initial email used to recruit potential stakeholders, and Appendix B presents the reminder email sent seven days later to non-respondents to the initial email. Ten days after the initial email was sent, phone outreach was initiated to non-respondents.

Table 1 lists the 20 panel members who agreed to participate (two-thirds of those contacted). The expert panel included six survey sponsors, five survey experts, six patient experience advocates, and three federal stakeholders. The majority were female (65%) and 65 or older (50%). Most of the panel members worked on either the West Coast (*n* = 9) or the East Coast (*n* = 7), while 2 were in the Midwest, 1 in the Mid-Atlantic, and 1 in the Southeast. Panel members were recruited for their knowledge and expertise at the local and national levels, not unique to their region of the country. Participating panel members who were eligible for compensation received $1500 for their time.

**Table 1:** Individuals providing input on the CAHPS ambulatory surveys

Name	Affiliation (Location)	Type of stakeholder
Taroon Amin	Centers for Medicare & Medicaid Services/Center for Medicare & Medicaid Innovation (Washington, DC)	Federal
Rachel DuPré Brodie	Purchaser Business Group on Health (San Francisco, CA)	Expert
Elizabeth Goldstein	Centers for Medicare & Medicaid Services /Center for Medicare (Washington, DC)	Federal
Daniel Green	Centers for Medicare & Medicaid Services/Center for Clinical Standards & Quality (Washington, DC)	Federal
Katherine Haynes	California Healthcare Foundation (Oakland, CA)	Advocate
Beverley H. Johnson	Institute for Family and Patient-Centered Care (McLean, VA)	Advocate
Lance Lang	California Health Policy Strategies, LLC (Sacramento, CA)	Sponsor
Rita Mangione-Smith	Kaiser Foundation Health Plan of Washington (Seattle, WA)	Expert
Elizabeth McGlynn	Kaiser Permanente Center for Effectiveness & Safety Research (Pasadena, CA)	Expert
Sheila Moroney	The Patient Revolution (St. Paul, MN)	Advocate
Charleen Mikail	ChapCare/AltaMed (Los Angeles, CA)	Sponsor
Amy Minnich	Geisinger (Danville, PA)	Sponsor
Armando Nahum	Patients for Patient Safety, and MedStar Institute for Quality and Safety (Atlanta, GA)	Advocate
Robert Parrish	Future Directions in Health Care (Ferndale, MI)	Advocate
Barbra Rabson	Massachusetts Health Quality Partners (Belmont, MA)	Sponsor
Dana Gelb Safran	National Quality Forum (Boston, MA)	Expert
Sarah Shih	National Committee for Quality Assurance (Washington, DC)	Expert
Samuel Skootsky	UCLA Faculty Practice Group (Los Angeles, CA)	Sponsor
S. Monica Soni	California Department of Health Care Services and Covered California (Washington, DC)	Sponsor
Ted Von Glahn	Independent Consultant (San Rafael, CA)	Advocate

### Modified appropriateness method

The RAND/UCLA Appropriateness Method involves two rating rounds and a discussion phase between the two rounds [14]. ExpertLens™, an online platform for modified Delphi studies [13, 15–17], was used to gather input about potential changes to the HP 5.0/5.1 and CG 3.0/3.1 surveys. The ExpertLens™ software collects information from non-collocated participants anonymously and iteratively, includes threaded discussion boards, and is cost-efficient. Like in-person expert panels, an experienced discussion facilitator was used to encourage discussion, solicit comments from all participants, and ensure that a subset of participants did not dominate the conversation.

There were three phases of data collection.

### Phase 1

**Fig. 1:**
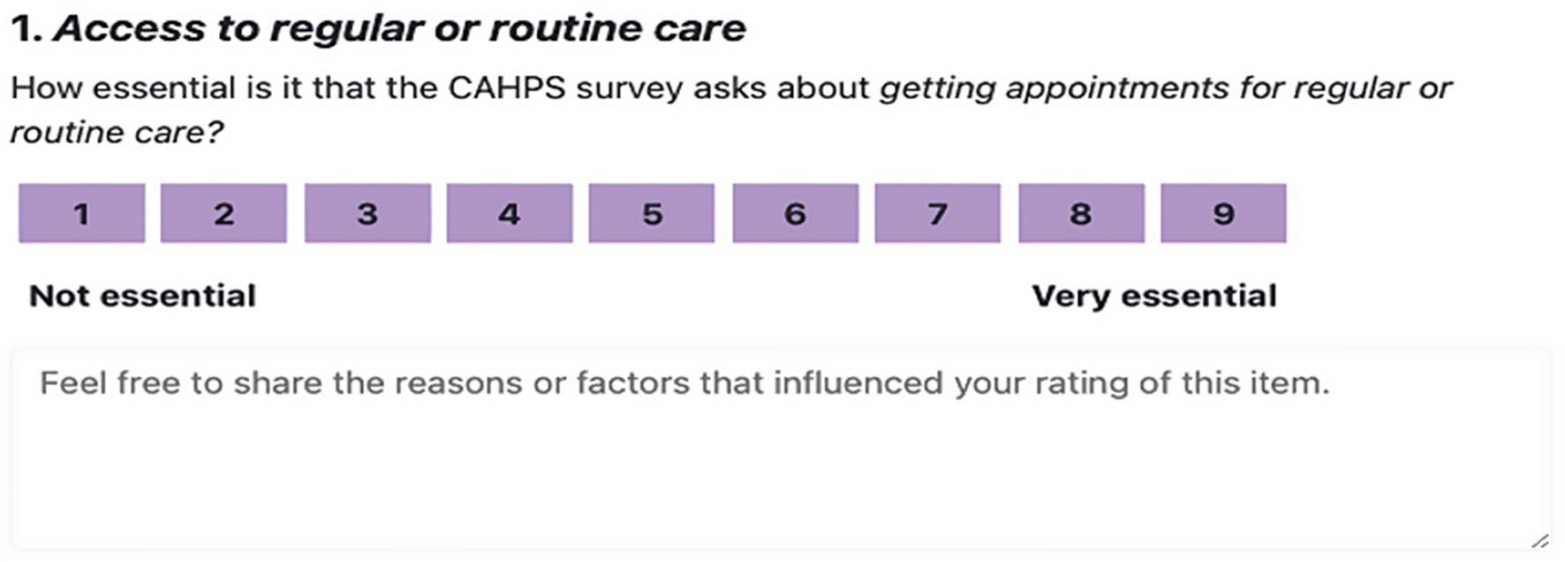
Example question asked of panelists

Initial ratings were obtained from panel members and their assessments about whether CAHPS ambulatory survey items should be required (core) or optional (supplemental). Panel members rated the extent to which 46 item topics (see Appendix C) were essential on a 9-point scale, ranging from “not essential” (1–3) to “optional” (4–6) to “required” (7–9). They had the option to share their rationale for each rating. Figure 1 provides an example question.

The following instructions preceded the questions:

“We ask you to rate each existing survey topic on a scale from 1 **(Not Essential)** to 9 **(Very Essential)**. We want you to interpret the rating scores as follows:

- Scores of 1 to 3 indicate the topic should *not* be included in a CAHPS survey of health plans, clinicians, or group practices.
- Scores of 4 to 6 indicate the topic should be optional in a CAHPS survey of health plans, clinicians, or group practices.
- Scores of 7 to 9 indicate the topic should be required in a CAHPS survey of health plans, clinicians, or group practices.

We encourage you to use all values within the scoring range (from ‘1’ to ‘9’) when rating the survey topics. Remember, you have three values within the range of ‘not be included,’ ‘optional’ and ‘required’ ratings of survey topics.

Panelists were also asked to suggest topics that should be added to the survey.”

### Phase 2

**Fig. 2:**
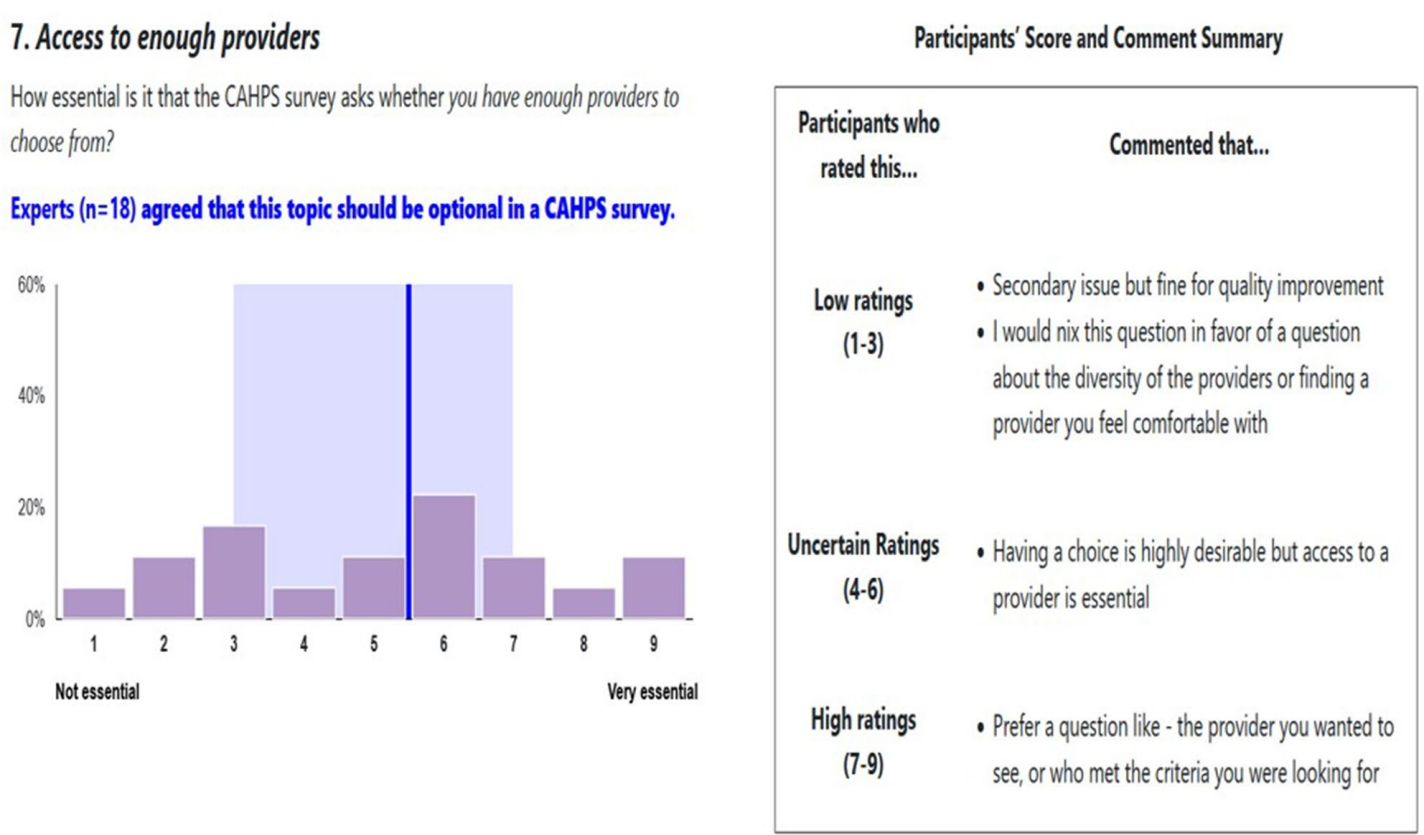
Example summary of results provided to panelists in Phase 2

Over the course of five subsequent days, panel members reviewed and anonymously discussed Phase 1 results on a discussion board with a threaded structure and a moderator. Asynchronous discussion facilitates engagement across time zones. The discussion board was used to encourage an exchange of ideas and clarify the rationale behind comments. Panel members reviewed the Phase 1 results, including one chart and one table for each topic. Each chart displayed the panel members’ ratings, median rating, interquartile range, and the distribution of ratings by the panelists. Each table provided a summary of the comments from participants in Phase 1, as produced by a moderator (Fig. 2). Panel members commented on ratings and engaged in discussion (including discussion of new topics) via a panel discussion board. Panel members were identified on the discussion board by their panelist identification number, not their name.

### Phase 3

In the final 12 days, panel members reviewed the Phase 2 discussion and could modify their Phase 1 ratings or confirm they want them to remain the same (subsequent ratings). Panelists were asked to explain why their ratings changed or stayed the same.

### Analysis plan

The reliability of the initial and subsequent expert 1–9 ratings of the 46 items was estimated using a mixed-effects analysis of variance model that partitions the main effects of items, experts, and the interaction between experts and items [18]. A reliability of 0.70 is considered acceptable for research [19]. The intraclass correlation represents the estimated reliability for a single rater. The agreement for the three categories of topic ratings (not essential (1–3 rating), optional (4–6 rating), and required (7–9 rating)) was estimated using free marginal kappa [20, 21]. A common rule of thumb for interpreting kappa is that 0.21–0.40 indicates fair agreement, 0.41–0.60 moderate agreement, 0.61–0.80 substantial agreement, and 0.81 or above almost perfect agreement [22].

Next, means and standard deviations for the 46 essential ratings on the 1–9 response scale are reported, after dichotomizing them by coding scores of 7–9 as “1” for “*essential*” and scores of 1–6 as “0” for “*not essential*.” Finally, the input received in verbatim comments by the experts is summarized.

## Results

### Reliability of expert essentialness ratings

Reliability estimates for the initial essentialness ratings of the 46 items were 0.63 (intraclass correlation = 0.08) for the 1–9 ratings and 0.62 (intraclass correlation = 0.08) for the dichotomized (i.e., 7–9 coded as 1 and 1–6 coded as 0) ratings. The overall agreement was 55% for the three-category scoring, and the free-marginal kappa was 0.33 (95% CI = 0.26 to 0.39).

As expected, reliability increased from the initial round to the final round of ratings. Reliability estimates for the final round of ratings were 0.70 (intraclass correlation = 0.10) for the 1–9 expert ratings and 0.62 (intraclass correlation = 0.08) for the dichotomized ratings. The overall agreement was 56% for the three-category scoring, and the free-marginal kappa was 0.35 (95% CI = 0.27 to 0.42).

### Distribution of essentialness ratings

Table 2 shows the means and standard deviations for the initial essentialness ratings for the 46 items. The means for the 1–9 expert ratings ranged from 5.1 (ask whether you have enough providers to choose from) to 8.4 (ask whether your health care provider shows respect for what you say). The average essential rating across the 46 items was 6.6 for panel members who were patient experience advocates, 7.0 for survey experts, 7.1 for survey sponsors, and 8.0 for federal stakeholders. The mean ratings did not differ significantly between the stakeholder subgroups: F (3,16 degrees of freedom) = 1.14, *p* = 0.3618).

**Table 2:** Means and standard deviations for round 1 ratings of essentialness of 47 items

Item	Mean (SD) 1-9 Scale	Mean (SD) 0-1 Score	Number of Respondents			
Essential to ask about whether you have enough providers to choose from?	5.11 (2.42)	0.28 (0.46)	18			
Essential to ask whether someone asked if you had concerns about cost of care, tests, or treatment?	5.59 (2.08)	0.29 (0.47)	17			
Essential to ask about whether appointments can be scheduled via email or a patient portal?	5.83 (2.31)	0.33 (0.49)	18			
Essential to ask about the ease of filling out forms or paperwork from your health plan?	5.42 (2.41)	0.37 (0.50)	19			
Essential to ask about whether someone asked if you had concerns about cost of prescription medication?	6.24 (2.05)	0.47 (0.51)	17			
Essential to ask about whether you can reach the health plan customer service by email or a patient portal?	5.82 (2.86)	0.47 (0.51)	17			
Essential to ask about ease of getting information from your health plan website or patient portal?	5.94 (2.16)	0.53 (0.51)	17			
Essential to ask about whether provider office gives you results of blood test, x-ray or other test?	6.61 (2.09)	0.56 (0.51)	18			
Essential to ask about your health care provider has access to your medical record during appointments?	6.60 (2.82)	0.60 (0.50)	20			
Essential to ask about the ease of getting care from specialists?	7.06 (2.36)	0.61 (0.50)	18			
Essential to ask about getting answers to medical questions during regular hours?	6.83 (1.58)	0.61 (0.50)	18			
Essential to ask about whether someone from your provider office asks about prescribed medication you are taking?	7.06 (1.86)	0.61 (0.50)	18			
Essential to ask about whether your health care provider encourages you to ask questions?	6.83 (2.56)	0.61 (0.50)	18			
Essential to ask about whether clerks and reception staff were courteous and helpful?	6.68 (2.21)	0.63 (0.50)	19			
Essential to ask about getting urgent care during regular hours?	7.12 (2.06)	0.65 (0.49)	17			
Essential to ask about whether clerks and reception staff treated you with respect?	6.82 (2.24)	0.65 (0.49)	17			
Essential to ask whether you would recommend your primary care provider to family and friends?	6.88 (2.42)	0.65 (0.49)	17			
Essential to ask for a 0 to 10 rating of your care from specialists?	6.65 (2.29)	0.65 (0.49)	17			
Essential to ask about whether you would recommend your health plan to family or friends?	6.76 (2.41)	0.65 (0.49)	17			
Essential to ask about getting appointments without long waits	6.94 (2.75)	0.67 (0.49)	18			
Essential to ask about whether you can reach your health care provider by email or a patient portal?	6.94 (1.86)	0.67 (0.49)	18			
Essential to ask about getting answers to medical questions during evening and weekends?	6.83 (1.58)	0.67 (0.49)	18			
Essential to ask about whether your health care provider understands your symptoms?	6.61 (2.82)	0.67 (0.49)	18			
Essential is it that the CAHPS survey asks for a 0 to 10 rating of your primary health care provider?	7.06 (2.43)	0.69 (0.48)	16			
Essential to ask about ease of getting help to navigate care from different providers or types of care?	6.82 (2.07)	0.71 (0.47)	17			
Essential to ask about the ease of making an appeal when a health care service is denied?	7.24 (1.52)	0.71 (0.47)	17			
Essential to ask about whether you have a primary care provider?	7.06 (2.26)	0.72 (0.46)	18			
Essential to ask a 0 to 10 rating of the health plan?	7.24 (2.34)	0.76 (0.44)	17			
Essential to ask about the availability of care by phone or video?	6.94 (1.86)	0.78 (0.43)	18			
Essential to ask about results of a blood test, x-ray or other medical test received without a long wait?	6.67 (2.74)	0.78 (0.43)	18			
Essential to ask for a 0 to 10 rating of all your health care?	7.21 (2.51)	0.79 (0.42)	19			
Essential to ask about whether your provider explains results of blood test, x-ray or other medical test?	7.81 (1.56)	0.81 (0.40)	16			
Essential to ask about whether your health plan customer service staff were courteous and respectful?	7.00 (1.75)	0.81 (0.40)	16			
Essential to ask about whether your provider spends enough time with you during an appointment?	7.18 (1.67)	0.82 (0.39)	17			
Essential to ask about whether you received clear information in advance on amount you would pay for care?	6.88 (1.65)	0.82 (0.39)	17			
Essential to ask about the ease of getting information from your health plan customer service?	7.47 (1.42)	0.82 (0.39)	17			
Essential to ask about getting urgent care during evenings and weekends?	7.11 (2.42)	0.83 (0.38)	18			
Essential to ask about you are able to find a specialist when needed?	7.61 (1.97)	0.83 (0.38)	18			
Essential to ask about the ease of getting needed care, tests or treatment?	7.88 (2.03)	0.88 (0.33)	17			
Essential to ask whether health care provider knows important information about your medical history?	7.82 (1.38)	0.88 (0.33)	17			
Essential to ask about whether you were treated in an unfair or insensitive way by providers or staff?	7.71 (2.05)	0.88 (0.33)	17			
Essential to ask about whether your health care provider listens carefully to you?	8.06 (1.16)	0.89 (0.32)	18			
Essential to ask about provider is knowledgeable about your ongoing or chronic health conditions?	7.78 (1.90)	0.94 (0.24)	18			
Essential to ask about whether your provider explains things in a way that is easy to understand?	8.28 (0.89)	0.94 (0.24)	18			
Essential to ask about whether your health care provider shows respect for what you have to say?	8.39 (1.09)	0.94 (0.24)	18			
Essential to ask about getting regular or routine care appointments	8.00 (1.84)	0.95 (0.22)	20			

Note: 1 (Not Essential) to 9 (Very Essential) scale with scores of 1-3 used for a topic that should not be included, 4-6 used for a topic that should be optional, and 7-9 for a topic that should be required in a CAHPS survey of health plans, clinicians, or group practices. The 1-9 scores were dichotomized (7-9 coded as 1 and 1-6 coded as 0) to produce the 0-1 score

Table 3 shows the means for the 0 (non-essential) versus 1 (essential) coding of the initial and final ratings. Initial ratings indicated that over 50% of panel members rated 40 of the 46 items essential. Initial means ranged from 0.28 (whether you have enough providers to choose from) to 0.95 (getting regular or routine care appointments). Final ratings showed that over 50% of panelists rated 40 of the 46 items to be essential (7–9). The means for the final ratings ranged from 0.21 (whether you have enough providers to choose from) to 0.95 (getting regular or routine care appointments).

**Table 3:** Means (Standard Deviations) of initial and final essentialness ratings

Item	Initial	Final
	Mean (SD) [*N*] 0-1 Score	Mean (SD) [*N*] 0-1 Score
Essential to ask about whether you have enough providers to choose from?	0.28 (0.46) [18]	0.21 (0.42) [19]
Essential to ask whether someone asked if you had concerns about cost of care, tests, or treatment?	0.29 (0.47) [17]	0.26 (0.45) [19]
Essential to ask about whether appointments can be scheduled via email or a patient portal?	0.33 (0.49) [18]	0.32 (0.48) [19]
Essential to ask about the ease of filling out forms or paperwork from your health plan?	0.37 (0.50) [19]	0.37 (0.50) [19]
Essential to ask about whether someone asked if you had concerns about cost of prescription medication?	0.47 (0.51) [17]	0.44 (0.51) [18]
Essential to ask about whether you can reach health plan customer service by email or a patient portal?	0.47 (0.51) [17]	0.47 (0.51) [17]
Essential to ask about ease of getting information from your health plan website or patient portal?	0.53 (0.51) [17]	**0.61** (0.50) [18]
Essential to ask about whether provider office gives you results of blood test, x-ray or other test?	0.56 (0.51) [18]	0.53 (0.51) [17]
Essential to ask about your health care provider has access to your medical record during appointments?	0.60 (0.50) [20]	**0.70** (0.47) [20]
Essential to ask about the ease of getting care from specialists?	0.61 (0.50) [18]	0.58 (0.51) [19]
Essential to ask about getting answers to medical questions during regular hours?	0.61 (0.50) [18]	0.67 (0.50) [18]
Essential to ask about whether someone from your provider office asks about prescribed medication you are taking?	0.61 (0.50) [18]	**0.72** (0.49) [18]
Essential to ask about whether your health care provider encourages you to ask questions?	0.61 (0.50) [18]	**0.78** (0.43) [18]
Essential to ask about whether clerks and reception staff were courteous and helpful?	0.63 (0.50) [19]	0.68 (0.48) [19]
Essential to ask about getting urgent care during regular hours?	0.65 (0.49) [17]	0.79 (0.42) [19]
Essential to ask about whether clerks and reception staff treated you with respect?	0.65 (0.49) [17]	0.67 (0.49) [18]
Essential to ask whether you would recommend your primary care provider to family and friends?	0.65 (0.49) [17]	0.61 (0.50) [18]
Essential to ask for a 0 to 10 rating of your care from specialists?	0.65 (0.49) [17]	**0.56** (0.51) [18]
Essential to ask about whether you would recommend your health plan to family or friends?	0.65 (0.49) [17]	0.67 (0.49) [18]
Essential to ask about getting appointments without long waits	0.67 (0.49) [18]	0.63 (0.50) [19]
Essential to ask about whether you can reach your health care provider by email or a patient portal?	0.67 (0.49) [18]	0.68 (0.48) [19]
Essential to ask about getting answers to medical questions during evening and weekends?	0.67 (0.49) [18]	**0.58** (0.51) [19]
Essential to ask about whether your health care provider understands your symptoms?	0.67 (0.49) [18]	0.72 (0.46) [18]
Essential is it that the CAHPS survey asks for a 0 to 10 rating of your primary health care provider?	0.69 (0.48) [16]	0.71 (0.47) [17]
Essential to ask about ease of getting help to navigate care from different providers or types of care?	0.71 (0.47) [17]	0.78 (0.43) [18]
Essential to ask about the ease of making an appeal when a health care service is denied?	0.71 (0.47) [17]	**0.88** (0.33) [17]
Essential to ask about whether you have a primary care provider?	0.72 (0.46) [18]	0.74 (0.45) [19]
Essential to ask a 0 to 10 rating of the health plan?	0.76 (0.44) [17]	0.76 (0.44) [17]
Essential to ask about the availability of care by phone or video?	0.78 (0.43) [18]	0.84 (0.37) [19]
Essential to ask about results of a blood test, x-ray or other medical test received without a long wait?	0.78 (0.43) [18]	0.79 (0.42) [19]
Essential to ask for a 0 to 10 rating of all your health care?	0.79 (0.42) [19]	0.72 (0.46) [18]
Essential to ask about whether your provider explains results of blood test, x-ray or other medical test?	0.81 (0.40) [16]	0.79 (0.42) [19]
Essential to ask about whether your health plan customer service staff were courteous and respectful?	0.81 (0.40) [16]	**0.89** (0.32) [18]
Essential to ask about whether your provider spends enough time with you during an appointment?	0.82 (0.39) [17]	**0.74** (0.45) [19]
Essential to ask about whether you received clear information in advance on amount you would pay for care?	0.82 (0.39) [17]	0.76 (0.44) [17]
Essential to ask about the ease of getting information from your health plan customer service?	0.82 (0.39) [17]	**0.94** (0.24) [18]
Essential to ask about getting urgent care during evenings and weekends?	0.83 (0.38) [18]	0.78 (0.43) [18]
Essential to ask about you are able to find a specialist when needed?	0.83 (0.38) [18]	0.79 (0.42) [19]
Essential to ask about the ease of getting needed care, tests or treatment?	0.88 (0.33) [17]	0.84 (0.37) [19]
Essential to ask whether health care provider knows important information about your medical history?	0.88 (0.33) [17]	0.89 (0.32) [19]
Essential to ask about whether you were treated in an unfair or insensitive way by providers or staff?	0.88 (0.33) [17]	0.95 (0.23) [19]
Essential to ask about whether your health care provider listens carefully to you?	0.89 (0.32) [18]	0.89 (0.32) [19]
Essential to ask about provider is knowledgeable about your ongoing or chronic health conditions?	0.94 (0.24) [18]	**0.79** (0.42) [19]
Essential to ask about whether your provider explains things in a way that is easy to understand?	0.94 (0.24) [18]	0.94 (0.24) [18]
Essential to ask about whether your health care provider shows respect for what you have to say?	0.94 (0.24) [18]	0.95 (0.23) [19]
Essential to ask about getting regular or routine care appointments	0.95 (0.22) [20]	0.95 (0.22) [20]

Note: The 0-1 score is a dichotomization of the 1 (Not Essential) to 9 (Very Essential) scale to separate scores that represent a required topic (i.e., rated 7-9 on the 1-9 scale, so recoded to 1) versus those rated optional or should not be included (i.e., rated 1-6 on the 1-9 scale, so recoded to 0). Bold is used to denote changes of 0.08 (absolute value) or larger from initial to final essentialness ratings

Bolded numbers in Table 3 denote large changes (0.08 or larger) from initial to final essentialness ratings. There were large increases in the rated essentialness of seven items:

1. Ease of getting information from your health plan website or patient portal;
2. Health care provider has access to your medical recording during appointments;
3. Someone from your provider’s office asks about prescribed medications you are taking;
4. Health care provider encourages you to ask questions;
5. Ease of making an appeal when a health care service is denied;
6. Health care plan customer service staff were courteous and respectful;
7. Ease of getting information from your health plan customer service.

There were noteworthy (0.08 or larger) decreases from the initial to subsequent ratings for the essentialness ratings of 4 items:

1. 0 to 10 rating of your care from specialists;
2. Getting answers to medical questions during evenings and weekends;
3. Provider is knowledgeable about your ongoing or chronic health conditions.
4. Provider spends enough time with you during an appointment.

Table 4 gives the rank order of the final ratings.

**Table 4:** Rank order of final essentialness ratings

Item	Mean (SD) [*N*] 0-1 Score
Essential to ask about whether you have enough providers to choose from?	0.21 (0.42) [19]
Essential to ask whether someone asked if you had concerns about cost of care, tests, or treatment?	0.26 (0.45) [19]
Essential to ask about whether appointments can be scheduled via email or a patient portal?	0.32 (0.48) [19]
Essential to ask about the ease of filling out forms or paperwork from your health plan?	0.37 (0.50) [19]
Essential to ask about whether someone asked if you had concerns about cost of prescription medication?	0.44 (0.51) [18]
Essential to ask about whether you can reach health plan customer service by email or a patient portal?	0.47 (0.51) [17]
Essential to ask about whether provider office gives you results of blood test, x-ray or other test?	0.53 (0.51) [17]
Essential to ask for a 0 to 10 rating of your care from specialists?	0.56 (0.51) [18]
Essential to ask about getting answers to medical questions during evening and weekends?	0.58 (0.51) [19]
Essential to ask about the ease of getting care from specialists?	0.58 (0.51) [19]
Essential to ask about ease of getting information from your health plan website or patient portal?	0.61 (0.50) [18]
Essential to ask whether you would recommend your primary care provider to family and friends?	0.61 (0.50) [18]
Essential to ask about getting appointments without long waits	0.63 (0.50) [19]
Essential to ask about whether clerks and reception staff treated you with respect?	0.67 (0.49) [18]
Essential to ask about whether you would recommend your health plan to family or friends?	0.67 (0.49) [18]
Essential to ask about getting answers to medical questions during regular hours?	0.67 (0.50) [18]
Essential to ask about whether clerks and reception staff were courteous and helpful?	0.68 (0.48) [19]
Essential to ask about whether you can reach your health care provider by email or a patient portal?	0.68 (0.48) [19]
Essential to ask about your health care provider has access to your medical record during appointments?	0.70 (0.47) [20]
Essential to ask about whether someone from your provider office asks about prescribed medication you are taking?	0.72 (0.49) [18]
Essential is it that the CAHPS survey asks for a 0 to 10 rating of your primary health care provider?	0.71 (0.47) [17]
Essential to ask about whether your health care provider understands your symptoms?	0.72 (0.46) [18]
Essential to ask for a 0 to 10 rating of all your health care?	0.72 (0.46) [18]
Essential to ask about whether you have a primary care provider?	0.74 (0.45) [19]
Essential to ask about whether your provider spends enough time with you during an appointment?	0.74 (0.45) [19]
Essential to ask a 0 to 10 rating of the health plan?	0.76 (0.44) [17]
Essential to ask about whether you received clear information in advance on amount you would pay for care?	0.76 (0.44) [17]
Essential to ask about whether your health care provider encourages you to ask questions?	0.78 (0.43) [18]
Essential to ask about ease of getting help to navigate care from different providers or types of care?	0.78 (0.43) [18]
Essential to ask about getting urgent care during evenings and weekends?	0.78 (0.43) [18]
Essential to ask about getting urgent care during regular hours?	0.79 (0.42) [19]
Essential to ask about results of a blood test, x-ray or other medical test received without a long wait?	0.79 (0.42) [19]
Essential to ask about whether your provider explains results of blood test, x-ray or other medical test?	0.79 (0.42) [19]
Essential to ask about you are able to find a specialist when needed?	0.79 (0.42) [19]
Essential to ask about provider is knowledgeable about your ongoing or chronic health conditions?	0.79 (0.42) [19]
Essential to ask about the ease of getting needed care, tests or treatment?	0.84 (0.37) [19]
Essential to ask about the availability of care by phone or video?	0.84 (0.37) [19]
Essential to ask about the ease of making an appeal when a health care service is denied?	0.88 (0.33) [17]
Essential to ask about whether your health plan customer service staff were courteous and respectful?	0.89 (0.32) [18]
Essential to ask whether health care provider knows important information about your medical history?	0.89 (0.32) [19]
Essential to ask about whether your health care provider listens carefully to you?	0.89 (0.32) [19]
Essential to ask about the ease of getting information from your health plan customer service?	0.94 (0.24) [18]
Essential to ask about whether your provider explains things in a way that is easy to understand?	0.94 (0.24) [18]
Essential to ask about whether you were treated in an unfair or insensitive way by providers or staff?	0.95 (0.23) [19]
Essential to ask about whether your health care provider shows respect for what you have to say?	0.95 (0.23) [19]
Essential to ask about getting regular or routine care appointments	0.95 (0.22) [20]

Note: The 0-1 score is a dichotomization of the 1 (Not Essential) to 9 (Very Essential) scale to separate scores that represent a required topic (i.e., rated 7-9 on the 1-9 scale, so recoded to 1) versus those rated optional or should not be included (i.e., rated 1-6 on the 1-9 scale, so recoded to 0)

### Expert verbatim input on different content areas

#### Access to care

In the discussion round, experts identified access to care as an essential element of healthcare, with seven experts across sections referring to access as “foundational,” “table stakes,” “critical,” and “essential.” Some experts suggested that the CAHPS survey may not capture patients who are unable to schedule an appointment successfully. However, eligibility for the health plan sample is based on enrollment, not utilization. The CAHPS health plan survey 5.0 and 5.1 include relevant access questions: (1) In the last 12 months when you needed care right away, how often did you get care as soon as needed? (2) In the last 12 months, how often was it easy to get the care, tests, or treatment you needed?

The rank order of the essentialness of access items were (1) getting regular or routine care appointments (95% of experts rated this to be essential in both rounds); (2) ease of getting needed care, tests or treatment (88% in round 1 and 84% in round 2), (3) able to find a specialist when needed (83%, 79%); (4) getting urgent care during evenings and weekends (83%, 78%); (5) availability of care by phone or video (78%, 84%); (6) reach your health care provider by email or a patient portal (67%, 68%); (7) getting answers to medical questions during evening and weekends (67%, 58%); (8) getting appointments without long waits (67%, 63%), (9) getting urgent care during regular hours (65%, 79%), (10) getting answers to medical questions during regular hours (61%, 67%), 11) ease of getting care from specialists (61%, 58%), 12) ease of getting information from your plan website or patient portal (51%, 61%), 13) reach health plan customer service by email or patient portal (47%, 47%), and 14) appointments can be scheduled via email or patient portal (33%, 32%).

##### Regular or primary care

In the discussion, most panel members indicated that measuring whether patients have a primary care provider is essential. Two panel members highlighted that patients may not have a regular primary care provider or seek most of their regular healthcare from a specialist, and two others agreed with these comments. Six panel members agreed with defining “primary care provider.”^1^ for Topic 6 (“How essential is it that the CAHPS survey asks whether you have a primary care provider?”). One stakeholder defined a primary care provider as “a single point of contact for continuous, comprehensive, and coordinated care.” Note the wording of the second CAHPS Clinician and Group Survey 3.1 question: “Is this the provider you usually see if you need a check-up, want advice about a health problem, or get sick or hurt?”

Two panel members highlighted that access may not be within the provider’s control, primarily due to shortages of primary care providers. However, two other panel members noted that provider practices—including automatic return-to-care appointments and the number of providers can impact patient access to care. Five of the panel members emphasized the importance of allowing patients to choose a provider they prefer, rather than offering them a certain number of providers.

Three panel members mentioned primary care or staffing shortages when discussing access, and two indicated that assessing this in the CAHPS ambulatory surveys is essential. One stakeholder commented that staff shortages cannot be improved at the provider’s level and may be dependent on systemic or statewide advocacy.

Some panel members felt that the questions about getting care quickly and getting needed care should be more explicit: Topic 2 (getting appointments without long waits), Topic 3 (getting urgent care during regular hours), and Topic 4 (getting urgent care during evenings and weekends). A stakeholder asked, “What is the definition of ‘long’? It was then noted that long waits are not a problem in certain circumstances. One stakeholder asked if regular hours meant 8:00 a.m. to 5:00 p.m., Monday through Friday. Another mentioned that some primary care is offered as a Saturday clinic or evening hours once a week.

##### Specialist care

Experts who emphasized the importance of specialist care also discussed the unique barriers patients face in accessing it. One stakeholder specifically noted the importance of access to behavioral health care. Five panel members felt that topic 12, “How essential is it that the CAHPS survey asks about the ease of getting care from specialists?” needed clarification. Panel members suggested asking for specific and actionable information rather than a broad question on ease of access. Suggestions included asking about securing an appointment or whether the health plan could provide the patient with meaningful assistance in finding a specialist.

##### Telehealth

While recognizing the importance of telemedicine and e-portals, panelists cautioned about disparities in digital literacy and health literacy, and that the mere availability of electronic options does not guarantee quality of care.

#### Communication and coordination

Panel members highlighted that many of the questions in this section reflected the standard of care, and asking these questions could pinpoint critical issues related to continuity of care. Panel members repeatedly emphasized the importance of patients feeling understood, listened to, and respected by their providers as key to achieving better patient health outcomes and increasing patient satisfaction. All of the communication and caring items were rated as essential by most of the panel members (% for initial rating, % for final rating): (1) your health care provider shows respect for what you have to say (94%, 95%); (2) your provider explains things in a way that is easy to understand (94%, 94%); (3) provider is knowledgeable about your ongoing or chronic health conditions (94%, 79%), (4) your provider listens carefully to you (89%, 89%), (5) provider knows important information about your medical history (88%, 89%); (6) spends enough time with you during an appointment (82%, 74%); (7) explains results of blood test, x-ray or other medical test (81%, 79%); (8) understands your symptoms (67%, 72%); (9) encourages you to ask questions (61%, 78%); (10) someone from your provider’s office asks about prescribed medicines you are taking (61%, 72%); 11) provider has access to your medical record during appointments (60%, 70%); and 12) provider’s office gives you results of blood test, x-ray or other test (56%, 53%).

Panel members emphasized the quality of understanding over the availability of information. For example, three of the panel members highlighted that whether medical records were available to the provider may not be possible for the patient to know, and this information was generally less valuable than patients’ feeling that the provider understood their medical records. However, four of the panel members responded that sometimes patients can get this impression from providers asking questions already documented in their medical records, and that it would be essential to measure.

For Topic 19 (“How essential is it that the CAHPS survey asks whether your health care provider’s office gives you results of a blood test, x-ray or other medical test?”), nine of the panel members highlighted that the availability of test results to patients was critical. Still, the explanation of test results was more important. These panel members recommended asking whether the provider explained the results to the patient in an understandable way.

#### Interactions with staff and providers

The essentialness ratings of topics in this category (% for initial rating, % for final rating) were: (1) treated in an unfair or insensitive way by providers or staff (88%, 89%), and (2) clerks and reception staff were courteous and helpful (63%, 68%).

##### Staff

Panel members who emphasized the importance of these questions also highlighted the importance of patient trust, respect, and health equity. Some panelists suggested these interactions were indirectly linked to clinical outcomes and thus less critical as standalone items.

Four of the panel members agreed that collapsing emergency care and ambulatory care in topic 31 (“How essential is it that the CAHPS survey asks whether you were treated in an unfair or insensitive way by providers or staff at a clinic, emergency room or doctor’s office?”) would negatively affect the quality of information from this question. These panel members agreed that differentiating between settings would yield higher quality of information. Three panel members throughout this section specifically mentioned the impact of racism on patients in interactions with staff and providers.

##### Cost

The essentialness ratings were: (1) received clear information in advance on the amount you would pay for care (82%, 76%), (2) ease of making an appeal when a health care service is denied (71%, 88%), and (3) someone asked if you had concerns about the cost of care, tests, or treatment (29%, 26%).

Five of the panel members agreed that measuring whether providers offer payment assistance to patients was also important. Three of the panel members highlighted that while presenting cost estimates may increase transparency, this can be challenging for providers to produce due to the variability between health plans. One stakeholder responded to these concerns by pointing out that a good-faith estimate would still be a patient-focused initiative and increase transparency.

### Interactions with health plans

Three panelists felt that the prevalence of paper forms from health plans was insufficient to warrant inclusion of Topic 39 (“How essential is it that the CAHPS survey asks about the ease of filling out forms or paperwork from your health plan?”). The rise of online forms was noted. Only 37% of the panel members considered the ease of filling out forms or paperwork from your health plan essential in both rounds of ratings.

Panel members generally valued ease of information from customer service over ease of information from health plan websites and e-portals. One stakeholder noted that assessing customer service was likely the “single most important item regarding the plan.” Four panel members recommended asking generally about the ease with which patients can contact customer service instead of explicitly asking about email or e-portal. The essentialness rating for assessing whether health plan customer service staff were courteous and respectful was 81% in round 1 and 89% in round 2.

### Overall ratings

Essentialness ratings (% for initial rating, % for final rating) were: (1) 0 to 10 rating of all health care (79%, 72%), (2) 0 to 10 rating of the health plan (76%, 76%), (3) 0–10 rating of primary care provider (69%, 71%), (4) 0–10 rating of specialists (65%, 56%), (5) recommend your health plan to family or friends (65%, 56%), and (6) recommend your primary care provider to family and friends (65%, 61%). Three of the panel members who disagreed with including questions on global ratings highlighted a lack of actionability surrounding these questions. Two panel members recommended asking for an overall rating of the primary care team rather than the primary care provider. Three of the panel members commented that they preferred the “willingness to recommend” approach over a global rating. Four of the panel members noted that topic 45 (“How essential is it that the CAHPS survey asks for a 0 to 10 rating of the health plan?”) was essential or helpful for capturing the overall experience. One stakeholder commented that a lack of open-ended patient responses limited the actionability of the global rating questions.

### Topics missing from CAHPS ambulatory surveys

Table 5 provides verbatim suggestions about topics missing from the CAHPS ambulatory surveys. Some of the suggestions touch on issues that are actively being addressed in CAHPS VI, such as adding questions about unfair treatment (comments #2, 14, and 15), updating the About You section (comments #6 and 9), mental health care (comment #9), and maternity care (comment #15). There were suggestions to enhance the assessment of coordination of care/team-based care (comments #11, 17, and 18), finding a doctor the patient is comfortable with who speaks their language (comment #8), and availability of care in the preferred language (comment #20). “New areas” suggested include trust (comments #3, 7, 12, and 19), self-management and treating the whole person (comments #7, 9, 11, 16 and 18), patient safety (comments #4 and 13), continuity of care (comments #5, 11, and 12), claims processing (comment#10), including negatively worded items (comment #7), and having narrative items on all CAHPS surveys (comment #1).

**Table 5:** Verbatim topic recommendations stakeholders mentioned as missing from CAHPS ambulatory surveys

1	Development of a patient (subscriber) narrative item set, tailored as necessary, for every CAHPS survey, including the health plan survey.
2	Questions about discrimination or unfair treatment, specifying discrimination due to race, ethnicity, gender, sexual orientation, age, and disability at a minimum.
3	Questions about trust in one’s primary care provider--covering relationship-based care, reliability, honesty/truthfulness, and ability.
4	Questions relevant to ambulatory care settings focused on patient safety.
5	Questions addressing continuity of care over time across providers and episodes.
6	The gender questions in the “About You” sections of the surveys really need to be updated to include many more options than Male or Female.
7	As noted below, having negatively framed items is important to get a more holistic picture of the experience with a clinician. Gaps include items on trust (including possibly whether the clinician has earned or lost the patient’s trust in the past X months; but also other already-validated trust items); Gaps also include the patient feeling the clinician has (and acts on!) the key information about their life circumstances to provide recommendations that the patient is willing and able to follow. Another gap could be asking whether the patient ever felt that the clinician lacked the necessary expertise to help solve their health problem.
8	As noted, I think it is more important to ask whether people have difficulty finding a doctor they are comfortable with (who speaks their language, who looks like them, etc.) than it is to ask how easy it is to find a primary care physician in general. While I know there is a physician shortage in CA, particularly among primary care providers, the key to a good relationship is how comfortable patients feel with their physicians.
9	The following concepts are missing and/or have been raised by plans as topics they are interested in assessing with CAHPS: - Questions regarding patient’s access to the mental health of mental health care services. - Questions regarding whether the patient left the appointment with an understanding of what they needed to do next to maintain their health/care – CAHPS demographic or core questions on sexual and gender identity (SOGI); e.g., whether providers were respectful of gender identity and expression; whether they considered these factors when providing care. - Digital literacy: If there are questions about accessing services or information through electronic modes, measuring this concept may be useful.
10	Claims processing and the speed at which the health plan handled the patients’ claims.
11	Current or past survey questions do not represent continuity and coordination of care. Patients often lament that the fragmentation of services overly burdens them. Previous questions did not get to the heart of this issue for patients. “Did your team help you coordinate your care, including appointment access, medications, etc.” Question whether the provider/clinician took time to learn about the patient’s personal story, preferences, and values. Did the provider take time to know and understand you as a person?
12	Continuity of care trust/caring element/Integration of behavioral and physical health care. Confidence -- instead of “doctor understood your symptoms” If probing personal doctor --finding one/experience with one -- the continuity/duration of the relationship. Many people choose a plan because of existing personal doctor relationships; this subgroup’s experience may differ from those members who don’t have personal doctors upon plan entry. Distinguish the “health plan customer self-service” domain and consider the extent to which candidate Q. addresses experiences in that domain.
13	Patient safety should be the priority
14	I think there should be items that get at the consumer experience of structural racism or sexism in these healthcare settings. For example, asking if the consumer feels their race, ethnicity, or gender identity impacted the care they received from their primary care provider (specialty provider, customer service, etc.). It would also be interesting to know how often consumers report that their health care needs were met by their health plan (primary care provider, specialty providers, etc.) over the prior 12 months
15	I understand that CAHPS is developing a maternity-specific set of supplementary questions, and this will be an important addition. I hope the questions will address the comprehensiveness of maternity care, e.g., prenatal, labor and delivery, and postpartum; this means there would need to be hospital and physician/group questions. It would also be good to assess if a patient had other maternity providers, such as a doula or midwife. For the question about feeling mistreated, is there a way to ask about the reason? (For example, did you feel mistreated? And, if so, was it for any of these reasons? It’s a tricky question to ask and very subjective, but I think it deserves exploration.)
16	It would be informative to understand any other suggestions or gaps from the patient’s perspective- maybe a generic question about “other recommendations: how can my doctor assist to improve my health? same for Health Plan? Do you have any thoughts about including questions used on inpatient surveys, like how well the team works together? Increasing reliance on other team members, like care managers, is important to see how patients perceive alignment among the team.
17	It would be useful to expand care coordination and access issues, including prior authorization issues from a health plan.
18	Perhaps a question on whether your health plan or clinician helps you achieve your health goals (something that leads to proactive outreach and more personalized care). There isn’t much around team-based care (a very old-school focus on a single provider).
19	Questions about the primary care team and trust.
20	Availability of services in the language you prefer

In the Phase 2 discussion, a stakeholder commented, “There were no questions about access to behavioral health…it is now often provided by carve-outs …but CAHPS should include an assessment of integration and coordination between physical and behavioral health care, which is best accomplished through primary care.” Another stakeholder indicated that where they work, survey questions are used to assess whether patients were screened for mental or behavioral health by their primary care doctors, referred, and able to receive the care they needed in a timely manner.

## Discussion

Using a modified Delphi approach, this study systematically elicited input from 20 stakeholders to explore potential improvements to the CAHPS ambulatory surveys. It leveraged the ExpertLens™ online platform for remote participation and threaded discussions, allowing for broader input and more thoughtful consideration than traditional in-person methods. The reliability of expert ratings increased from the initial to the final round, suggesting that the Delphi process effectively refined opinions. The final round of ratings had acceptable reliability (0.70). Kappa estimates of agreement for the three categories of not essential, optional, and required (7–9 ratings) for the initial and final ratings were fair [22]. A larger sample of stakeholders would be needed to achieve higher reliability.

While the mean essentialness ratings did not differ by type of stakeholder (patient experience advocates, survey experts, survey sponsors, federal stakeholders), different viewpoints were expressed in narrative comments, such as whether patients observe medical records availability, the definition of long waits, and the actionability of global ratings. In addition, while stakeholders deemed most of the existing survey items essential, some shifts occurred between rounds. Increases in essentialness were noted for digital access (website/portal information), medication reconciliation, provider encouragement of questions, appeals process, and customer service interactions. Decreases were seen for specialist care ratings, weekend/evening medical question access, and provider knowledge of chronic conditions.

Access to care was a central theme, with experts emphasizing its importance. Discussions centered on capturing patients who couldn’t obtain appointments (not just those who could), defining “long waits,” and addressing primary care provider shortages. Specialist access, particularly behavioral health, was highlighted as needing more specific and actionable questions. The need to assess digital and health literacy was also raised.

Communication between providers and patients, as well as care coordination, was highly valued, with most items related to provider respect, listening, and clear explanations being rated as essential. The quality of understanding was deemed more important than simply having medical records available. A clear explanation of test results was also prioritized. Respectful treatment by staff and providers was considered crucial. Concerns about the impact of racism on patient interactions were raised. The panel suggested separating emergency care and ambulatory care settings in questions about unfair treatment.

Clear information about costs and the appeals process was considered necessary. Some panelists suggested including questions about payment assistance, while others acknowledged the challenges of providing cost estimates due to insurance variability. The ease of contacting customer service was prioritized over the usability of the website or portal. There was a discussion about the relevance of questions regarding paper forms in the digital age.

There was variation in perceptions of the global ratings (0–10 scale) and willingness to recommend items. Some panelists questioned the actionability of these ratings, while others found them valuable. A preference for rating the “primary care *team*” over the individual provider was suggested.

Topics to consider in revising the ambulatory surveys include: (1) Unfair treatment; (2) Mental health care integration and coordination; (3) Maternity care; (4) Finding a doctor who speaks the patient’s language; (5) Availability of care in one’s preferred language; (6) Trust; (7) Self-management and whole-person care; (8) Patient safety; (9) Continuity of care; (10) Enhanced assessment of care coordination and team-based care; and 11) Claims processing.

### Limitations

The study’s results are limited to the input of the survey sponsors, survey experts, patient experience advocates, and federal stakeholders who participated. Agreement among the stakeholders on the essentialness of topics was fair. The participating experts did not represent all relevant groups, such as patients from underrepresented backgrounds. Concerns have been raised about whether separate Delphi panels would yield the same results [23]. Finally, the way the rating task was described could have influenced the findings (framing bias).

## Conclusions

Despite concerns before the study that the survey was too long, the panel suggested minimal cuts and recommended additional items. The findings suggest potential revisions to existing items and expansion to capture patient experience better in a rapidly evolving healthcare landscape. Potential revisions to the CAHPS health plan and clinician and group surveys by the CAHPS consortium based on the stakeholder panel input will need to be vetted with existing users, such as the Centers for Medicare and Medicaid Services. Any new item wording will need to be evaluated using cognitive interviews with English-language and Spanish-language patients. The next step after cognitive interviews will be to conduct field tests to evaluate the psychometric properties of the revised survey instruments with patients and evaluate the extent to which results from existing versions of the surveys can be trended over time.

## Supplementary Information

Below is the link to the electronic supplementary material.

Supplementary Material 1

Supplementary Material 2

Supplementary Material 3

## Data Availability

All data included in this published article are available upon reasonable request.

## Footnotes

1 This is an example of how the stakeholders felt that they were supposed to wordsmith the survey topics presented to them—that is, they treated these as if they were the survey items.
