# Supplementary material for: Stakeholder input on the CAHPS ambulatory surveys
Source: J Patient Rep Outcomes. 2025 Dec 22;10:14. doi: 10.1186/s41687-025-00983-1 (PMC12834880; doi:10.1186/s41687-025-00983-1)
Supplement: Supplementary file 1 — Supplementary Material 1 [file 41687_2025_983_MOESM1_ESM.docx]

**Appendix A: Initial Invitation Email**

Subject: Invitation to Join Technical Expert Panel About Content of the Consumer Assessment of Healthcare Providers and Systems (CAHPS) Surveys

Dear [Recipient's Name],

CAHPS ambulatory care surveys play a crucial role in evaluating patient experiences with the care provided by health plans, group practices, Accountable Care Organizations, patient medical homes, and individual clinicians.

Because of your expertise in [insert relevant area or expertise], we hope you will participate as a member of our Technical Expert Panel (TEP) focused on refining the content of the CAHPS Health Plan and Clinician & Group Surveys. As a member of the TEP, you will provide invaluable feedback on current survey domains and suggestions on new topic areas via an electronic Delphi panel process.

Your insights and expertise will help shape the future direction of the CAHPS Health Plan and Clinician & Group Surveys, ensuring their continued relevance and effectiveness in capturing patient experiences. In appreciation of your participation and contribution, you will receive an honorarium of $1,500.

Key Details:

- **Panel Purpose**: To provide input on priorities for new and existing survey domains within the CAHPS Health Plan and Clinician & Group Surveys.

- **Panel Process**: Feedback on existing topics and nomination of new topics via a web-based panel tool. We anticipate up to three rounds of ratings of current CAHPS survey topics and submission of new topics. At most, this will require 3 hours of your time.

- **Honorarium**: $1,500 for participation in the panel.

Please let us know of your willingness to serve on this esteemed panel by Friday, May 3. Should you have any questions or require further information, please do not hesitate to contact [masked for peer review].

We look forward to the possibility of collaborating with you and benefiting from your expertise.
