# Supplementary material for: Stakeholder input on the CAHPS ambulatory surveys
Source: J Patient Rep Outcomes. 2025 Dec 22;10:14. doi: 10.1186/s41687-025-00983-1 (PMC12834880; doi:10.1186/s41687-025-00983-1)
Supplement: Supplementary file 2 — Supplementary Material 2 [file 41687_2025_983_MOESM2_ESM.docx]

**Appendix B: Follow-up email sent 7 days after the initial email to non-respondents**

Subject: Follow-up on Invitation to Join Technical Expert Panel About Content of the Consumer Assessment of Healthcare Providers and Systems (CAHPS) Surveys

Dear [Recipient's Name],

I’m reaching out to follow up on our recent invitation to participate in the AHRQ-funded Technical Expert Panel (TEP) focused on the content of the CAHPS Health Plan and Clinician & Group Surveys.

As a member of the TEP, the information you provide will inform updates to the CAHPS Health Plan and Clinician & Group Surveys, ensuring their continued relevance and effectiveness in capturing patient experiences.

Key Details:

- **Panel Purpose**: To provide input on priorities for new and existing survey domains within the CAHPS Health Plan and Clinician & Group Surveys.

- **Panel Process**: Feedback on existing topics and nomination of new topics via a web-based panel tool. We anticipate up to three rounds of ratings of current CAHPS survey topics and submission of new topics. At most, this will require 3 hours of your time.

- **Honorarium**: $1,500 for participation in the panel.

Please let us know of your willingness to serve on this esteemed panel by Friday, May 3. Should you have any questions or require further information, please do not hesitate to contact me at [masked for peer review].
