# Supplementary material for: Stakeholder input on the CAHPS ambulatory surveys
Source: J Patient Rep Outcomes. 2025 Dec 22;10:14. doi: 10.1186/s41687-025-00983-1 (PMC12834880; doi:10.1186/s41687-025-00983-1)
Supplement: Supplementary file 3 — Supplementary Material 3 [file 41687_2025_983_MOESM3_ESM.docx]

**Appendix C: Questions Asked of Panel members**

**1. Access to regular or routine care**

How essential is it that the CAHPS survey asks about *getting appointments for regular or routine care*?

1 2 3 4 5 6 7 8 9

Not Essential Very Essential

Should it be required or optional for a survey sponsor to include this topic in a CAHPS ambulatory care survey? Feel free to share why (TEXT BOX)

**2. Access to care without long waits**

How essential is it that the CAHPS survey asks about *getting appointments without long waits*?

1 2 3 4 5 6 7 8 9

Not Essential Very Essential

Should it be required or optional for a survey sponsor to include this topic in a CAHPS ambulatory care survey? Feel free to share why (TEXT BOX)

**3. Access to urgent care during regular hours**

How essential is it that the CAHPS survey asks about *getting urgent care during regular hours*?

1 2 3 4 5 6 7 8 9

Not Essential Very Essential

Should it be required or optional for a survey sponsor to include this topic in a CAHPS ambulatory care survey? Feel free to share why (TEXT BOX)

**4. Access to urgent care evenings and weekends**

How essential is it that the CAHPS survey asks about *getting urgent care during evenings and weekends*?

1 2 3 4 5 6 7 8 9

Not Essential Very Essential

Should it be required or optional for a survey sponsor to include this topic in a CAHPS ambulatory care survey? Feel free to share why (TEXT BOX)

**5. Access to electronic appointment scheduling**

How essential is it that the CAHPS survey asks whether *appointments can be scheduled via email or patient portal*?

1 2 3 4 5 6 7 8 9

Not Essential Very Essential

Should it be required or optional for a survey sponsor to include this topic in a CAHPS ambulatory care survey? Feel free to share why (TEXT BOX)

**6. Having a primary care provider**

How essential is it that the CAHPS survey asks whether *you have a primary care provider*?

1 2 3 4 5 6 7 8 9

Not Essential Very Essential

**7. Access to enough providers**

How essential is it that the CAHPS survey asks whether *you have enough providers to choose from*?

1 2 3 4 5 6 7 8 9

Not Essential Very Essential

Should it be required or optional for a survey sponsor to include this topic in a CAHPS ambulatory care survey? Feel free to share why (TEXT BOX)

**8. Access to telehealth care**

How essential is it that the CAHPS survey asks about the *availability of care by phone or video*?

1 2 3 4 5 6 7 8 9

Not Essential Very Essential

Should it be required or optional for a survey sponsor to include this topic in a CAHPS ambulatory care survey? Feel free to share why (TEXT BOX)

**9. Provider can be reached by email or a patient portal**

How essential is it that the CAHPS survey asks about whether *you can reach your health care provider by email or a patient portal?*

1 2 3 4 5 6 7 8 9

Not Essential Very Essential

Should it be required or optional for a survey sponsor to include this topic in a CAHPS ambulatory care survey? Feel free to share why (TEXT BOX)

**10. Access to needed care**

How essential is it that the CAHPS survey asks about the *ease of getting needed care, tests or treatment*?

1 2 3 4 5 6 7 8 9

Not Essential Very Essential

Should it be required or optional for a survey sponsor to include this topic in a CAHPS ambulatory care survey? Feel free to share why (TEXT BOX)

**11. Access to specialists when needed**

How essential is it that the CAHPS survey asks whether you *are able to find a specialist when needed*?

1 2 3 4 5 6 7 8 9

Not Essential Very Essential

Should it be required or optional for a survey sponsor to include this topic in a CAHPS ambulatory care survey? Feel free to share why (TEXT BOX)

**12. Ease of getting specialist care**

How essential is it that the CAHPS survey asks about the *ease of getting care* *from specialists*?

1 2 3 4 5 6 7 8 9

Not Essential Very Essential

Should it be required or optional for a survey sponsor to include this topic in a CAHPS ambulatory care survey? Feel free to share why (TEXT BOX)

**13. Asking medical questions during regular hours**

How essential is it that the CAHPS survey asks about *getting answers to medical questions during regular hours*?

1 2 3 4 5 6 7 8 9

Not Essential Very Essential

Should it be required or optional for a survey sponsor to include this topic in a CAHPS ambulatory care survey? Feel free to share why (TEXT BOX)

**14. Asking medical questions during evenings and weekends**

How essential is it that the CAHPS survey asks about *getting answers to medical questions during evening and weekends*?

1 2 3 4 5 6 7 8 9

Not Essential Very Essential

Should it be required or optional for a survey sponsor to include this topic in a CAHPS ambulatory care survey? Feel free to share why (TEXT BOX)

**15. Provider has medical record during appointment**

How essential is it that the CAHPS survey asks whether *your health care provider has access to your medical record during appointments*?

1 2 3 4 5 6 7 8 9

Not Essential Very Essential

Should it be required or optional for a survey sponsor to include this topic in a CAHPS ambulatory care survey? Feel free to share why (TEXT BOX)

**16. Provider knows medical history**

How essential is it that the CAHPS survey asks whether *your health care provider knows important information about your medical history*?

1 2 3 4 5 6 7 8 9

Not Essential Very Essential

Should it be required or optional for a survey sponsor to include this topic in a CAHPS ambulatory care survey? Feel free to share why (TEXT BOX)

**17. Provider knows about ongoing or chronic health conditions**

How essential is it that the CAHPS survey asks whether *your health care provider is* *knowledgeable about your ongoing or chronic health condition*s?

1 2 3 4 5 6 7 8 9

Not Essential Very Essential

Should it be required or optional for a survey sponsor to include this topic in a CAHPS ambulatory care survey? Feel free to share why (TEXT BOX)

**18. Provider understands symptoms**

How essential is it that the CAHPS survey asks whether *your health care provider understands your symptoms*?

1 2 3 4 5 6 7 8 9

Not Essential Very Essential

Should it be required or optional for a survey sponsor to include this topic in a CAHPS ambulatory care survey? Feel free to share why (TEXT BOX)

**19. Provider’s office gives you test results**

How essential is it that the CAHPS survey asks whether *your health care provider’s office gives you results of a blood test, x-ray or other medical test*?

1 2 3 4 5 6 7 8 9

Not Essential Very Essential

Should it be required or optional for a survey sponsor to include this topic in a CAHPS ambulatory care survey? Feel free to share why (TEXT BOX)

**20. Provider explains test results**

How essential is it that the CAHPS survey asks whether *your health care provider explains the results of your blood test, x-ray or other medical test*?

1 2 3 4 5 6 7 8 9

Not Essential Very Essential

Should it be required or optional for a survey sponsor to include this topic in a CAHPS ambulatory care survey? Feel free to share why (TEXT BOX)

**21. Test results received without long wait**

How essential is it that the CAHPS survey asks whether *results of a blood test, x-ray or other medical test are received without a long wait*?

1 2 3 4 5 6 7 8 9

Not Essential Very Essential

Should it be required or optional for a survey sponsor to include this topic in a CAHPS ambulatory care survey? Feel free to share why (TEXT BOX)

**22. Prescription medicines are documented**

How essential is it that the CAHPS survey asks whether *someone from your health care provider’s office asks about all the prescription medicines you are taking*?

1 2 3 4 5 6 7 8 9

Not Essential Very Essential

Should it be required or optional for a survey sponsor to include this topic in a CAHPS ambulatory care survey? Feel free to share why (TEXT BOX)

**23. Getting help to coordinate care**

How essential is it that the CAHPS survey asks about the *ease of getting help to navigate care from different providers or different types of care*?

1 2 3 4 5 6 7 8 9

Not Essential Very Essential

Should it be required or optional for a survey sponsor to include this topic in a CAHPS ambulatory care survey? Feel free to share why (TEXT BOX)

**24. Provider explanations are easy to understand**

How essential is it that the CAHPS survey asks whether *your health care provider explains things in a way that is easy to understand*?

1 2 3 4 5 6 7 8 9

Not Essential Very Essential

Should it be required or optional for a survey sponsor to include this topic in a CAHPS ambulatory care survey? Feel free to share why (TEXT BOX)

**25. Provider listens carefully**

How essential is it that the CAHPS survey asks whether *your health care provider listens carefully to you*?

1 2 3 4 5 6 7 8 9

Not Essential Very Essential

Should it be required or optional for a survey sponsor to include this topic in a CAHPS ambulatory care survey? Feel free to share why (TEXT BOX)

**26. Provider shows respect**

How essential is it that the CAHPS survey asks whether *your health care provider shows respect for what you have to say*?

1 2 3 4 5 6 7 8 9

Not Essential Very Essential

Should it be required or optional for a survey sponsor to include this topic in a CAHPS ambulatory care survey? Feel free to share why (TEXT BOX)

**27. Provider encourages questions**

How essential is it that the CAHPS survey asks whether *your health care provider encourages you to ask questions*?

1 2 3 4 5 6 7 8 9

Not Essential Very Essential

Should it be required or optional for a survey sponsor to include this topic in a CAHPS ambulatory care survey? Feel free to share why (TEXT BOX)

**28. Provider spends enough time**

How essential is it that the CAHPS survey asks whether *your health care provider spends enough time with you during an appointment*?

1 2 3 4 5 6 7 8 9

Not Essential Very Essential

Should it be required or optional for a survey sponsor to include this topic in a CAHPS ambulatory care survey? Feel free to share why (TEXT BOX)

**29. Courteous and helpful office staff**

How essential is it that the CAHPS survey asks whether *clerks and reception staff at a provider’s office were courteous and helpful*?

1 2 3 4 5 6 7 8 9

Not Essential Very Essential

Should it be required or optional for a survey sponsor to include this topic in a CAHPS ambulatory care survey? Feel free to share why (TEXT BOX)

**30. Respectful office staff**

How essential is it that the CAHPS survey asks whether *clerks and reception staff at a provider’s office treated you with respect*?

1 2 3 4 5 6 7 8 9

Not Essential Very Essential

Should it be required or optional for a survey sponsor to include this topic in a CAHPS ambulatory care survey? Feel free to share why (TEXT BOX)

**31. Unfair or insensitive treatment in health care setting**

How essential is it that the CAHPS survey asks whether *you were treated in an unfair or insensitive way by providers or staff at a clinic, emergency room or doctor’s office*?

1 2 3 4 5 6 7 8 9

Not Essential Very Essential

Should it be required or optional for a survey sponsor to include this topic in a CAHPS ambulatory care survey? Feel free to share why (TEXT BOX)

**32. Cost of care, tests, or treatment**

How essential is it that the CAHPS survey asks whether *someone from your health care provider’s office asked if you had concerns about cost of care, tests, or treatment*?

1 2 3 4 5 6 7 8 9

Not Essential Very Essential

Should it be required or optional for a survey sponsor to include this topic in a CAHPS ambulatory care survey? Feel free to share why (TEXT BOX)

**33. Information on cost of care, tests, or treatment**

How essential is it that the CAHPS survey asks whether *you received clear information in advance on the amount you would need to pay for care, tests, or treatment*?

1 2 3 4 5 6 7 8 9

Not Essential Very Essential

Should it be required or optional for a survey sponsor to include this topic in a CAHPS ambulatory care survey? Feel free to share why (TEXT BOX)

**34. Cost of prescription medication**

How essential is it that the CAHPS survey asks whether *someone from health care provider’s office asked if you had concerns about cost of prescription medication*?

1 2 3 4 5 6 7 8 9

Not Essential Very Essential

Should it be required or optional for a survey sponsor to include this topic in a CAHPS ambulatory care survey? Feel free to share why (TEXT BOX)

**35. Overall rating of health care**

How essential is it that the CAHPS survey asks for a *0 to 10 rating of all your health care*?

1 2 3 4 5 6 7 8 9

Not Essential Very Essential

Should it be required or optional for a survey sponsor to include this topic in a CAHPS ambulatory care survey? Feel free to share why (TEXT BOX)

**36. Overall rating of your primary health care provider**

How essential is it that the CAHPS survey asks for a *0 to 10 rating of your primary health care provider*?

1 2 3 4 5 6 7 8 9

Not Essential Very Essential

Should it be required or optional for a survey sponsor to include this topic in a CAHPS ambulatory care survey? Feel free to share why (TEXT BOX)

**37. Recommend your primary health care provider**

How essential is it that the CAHPS survey asks whether *you would recommend your primary care provider to family and friends*?

1 2 3 4 5 6 7 8 9

Not Essential Very Essential

Should it be required or optional for a survey sponsor to include this topic in a CAHPS ambulatory care survey? Feel free to share why (TEXT BOX)

**38. Overall rating of your care from specialists**

How essential is it that the CAHPS survey asks for a *0 to 10 rating of your care from specialists*?

1 2 3 4 5 6 7 8 9

Not Essential Very Essential

Should it be required or optional for a survey sponsor to include this topic in a CAHPS ambulatory care survey? Feel free to share why (TEXT BOX)

**39. Health plan paperwork**

How essential is it that the CAHPS survey asks about the *ease of filling out forms or paperwork from your health plan*?

1 2 3 4 5 6 7 8 9

Not Essential Very Essential

Should it be required or optional for a survey sponsor to include this topic in a CAHPS **health plan** survey? Feel free to share why (TEXT BOX)

**40. Health plan website or patient portal**

How essential is it that the CAHPS survey asks about the *ease of getting information from your health plan’s website or patient portal*?

1 2 3 4 5 6 7 8 9

Not Essential Very Essential

Should it be required or optional for a survey sponsor to include this topic in a CAHPS **health plan** survey? Feel free to share why (TEXT BOX)

**41. Health plan customer service**

How essential is it that the CAHPS survey asks about the *ease of getting information from your health plan’s customer service*?

1 2 3 4 5 6 7 8 9

Not Essential Very Essential

Should it be required or optional for a survey sponsor to include this topic in a CAHPS **health plan** survey? Feel free to share why (TEXT BOX)

**42. Customer service can be reached by email or a patient portal**

How essential is it that the CAHPS survey asks whether *you can reach your health plan’s customer service by email or a patient portal*?

1 2 3 4 5 6 7 8 9

Not Essential Very Essential

Should it be required or optional for a survey sponsor to include this topic in a CAHPS **health plan** survey? Feel free to share why (TEXT BOX)

**43. Courteous and respectful customer service staff**

How essential is it that the CAHPS survey asks whether *your health plan’s customer service staff were courteous and respectful*?

1 2 3 4 5 6 7 8 9

Not Essential Very Essential

Should it be required or optional for a survey sponsor to include this topic in a CAHPS **health plan** survey? Feel free to share why (TEXT BOX)

**44. Ease of appealing service denial**

How essential is it that the CAHPS survey asks about the *ease of making an appeal when a health care service is denied*?

1 2 3 4 5 6 7 8 9

Not Essential Very Essential

Should it be required or optional for a survey sponsor to include this topic in a CAHPS **health plan** survey? Feel free to share why (TEXT BOX)

**45. Overall rating of health plan**

How essential is it that the CAHPS survey asks *for a 0 to 10 rating of the health plan*?

1 2 3 4 5 6 7 8 9

Not Essential Very Essential

Should it be required or optional for a survey sponsor to include this topic in a CAHPS **health plan** survey? Feel free to share why (TEXT BOX)

**46. Recommend health plan**

How essential is it that the CAHPS survey asks whether *you would recommend your health plan to family or friends*?

1 2 3 4 5 6 7 8 9

Not Essential Very Essential

Should it be required or optional for a survey sponsor to include this topic in a CAHPS **health plan** survey? Feel free to share why (TEXT BOX)

**47. Topics Missing From CAHPS Surveys**

Are there topics you think are missing from CAHPS surveys of ambulatory care? Please enter any topics that should be required for a CAHPS survey of ambulatory care or a CAHPS health plan survey (TEXT BOX)

*Note: When the questions were asked again in the second and final round of ratings, each was followed by the question, “Did you change your rating from Round 1? Why or why not?*
